# Impact of Wild Loci on the Allergenic Potential of Cultivated Tomato Fruits

**DOI:** 10.1371/journal.pone.0155803

**Published:** 2016-05-16

**Authors:** Alessandra Ghiani, Nunzio D’Agostino, Sandra Citterio, Assunta Raiola, Riccardo Asero, Amalia Barone, Maria Manuela Rigano

**Affiliations:** 1 Department of Earth and Environmental Sciences, University of Milano-Bicocca, Milan, Italy; 2 Consiglio per la ricerca in agricoltura e l'analisi dell'economia agraria - Centro di ricerca per l'orticoltura, Pontecagnano Faiano, Italy; 3 Department of Agricultural Sciences, University of Naples "Federico II", Portici, Naples, Italy; 4 Allergy Unit, Clinica San Carlo, Paderno Dugnano, Milan, Italy; Mayo Clinic Arizona, UNITED STATES

## Abstract

Tomato (*Solanum lycopersicum*) is one of the most extensively consumed vegetables but, unfortunately, it is also able to induce allergic reactions. In the past, it has been shown that the choice of tomato cultivar significantly influenced the allergic reaction of tomato allergic subjects. In this study we investigated the allergenic potential of the cultivated tomato line M82 and of two selected lines carrying small chromosome regions from the wild species *Solanum pennellii* (i.e. IL7-3 and IL12-4). We evaluated the positive interactions of IgEs of allergic subjects in order to investigate the different allergenic potential of the lines under investigation. We used proteomic analyses in order to identify putative tomato allergens. In addition, bioinformatic and transcriptomic approaches were applied in order to analyse the structure and the expression profiles of the identified allergen-encoding genes. These analyses demonstrated that fruits harvested from the two selected introgression lines harbour a different allergenic potential as those from the cultivated genotype M82. The different allergenicity found within the three lines was mostly due to differences in the IgE recognition of a polygalacturonase enzyme (46 kDa), one of the major tomato allergens, and of a pectin methylesterase (34 kDa); both the proteins were more immunoreactive in IL7-3 compared to IL12-4 and M82. The observed differences in the allergenic potential were mostly due to line-dependent translational control or post-translational modifications of the allergens. We demonstrated, for the first time, that the introgression from a wild species (*S*. *pennellii*) in the genomic background of a cultivated tomato line influences the allergenic properties of the fruits. Our findings could support the isolation of favorable wild loci promoting low allergenic potential in tomato.

## Introduction

Tomato (*Solanum lycopersicum*) is one of the most consumed vegetables around the world (http://faostat.fao.org) and its raw or processed consumption increased over the last two decades also because it contains numerous nutritional and health-related compounds [1–3]. Unfortunately, tomato may be also involved in local (oral allergy syndrome) or systemic (urticaria/angioedema) allergic reactions [4]. Although some studies claim that the prevalence of tomato allergy ranges from 1.5% in Northern Europe up to more than 16% in Italy [3,4], other studies suggest much lower figures and show that most cases of tomato allergy are due to the co-recognition of the plant panallergen profilin [5–7].

To date, 27 potential tomato allergens, including different isoforms, have been reported and were published in different databases [4]. In particular, six tomato allergens were recognized by the International Union of Immunological Society (IUIS): profilin (Sola I 1), beta-fructofuranosidase (Sola I 2), non-specific lipid-transfer protein (nsLTP; Sola I 3), intracellular pathogenesis-related protein (Sola I 4), cyclophilin (Sola I 5) and nsLTP 7s (Sola I 6). In particular, Sola I 3 is an allergen still present in processed tomato products [3].

In previous studies it has been demonstrated that the allergenic potential of tomato fruits depends also on the cultivar under investigation [2,3,8]. Variable reaction patterns of patients' sera to tomato fruits from different cultivars have been described [9–11]. Moreover, when endogenous allergen concentrations have been characterised in different tomato cultivars, they have been found to vary significantly [3,12]. However, up to date, the characterization of the endogenous allergen content within new hybrids or varieties produced through traditional breeding has not been considered a priority also because usually allergic individuals avoid the allergenic food regardless of the used cultivar [13].

As for other crops, tomato improvement depends on the availability of genetic variability. The germplasm of cultivated tomato shows a reduced genetic variability as a consequence of its natural reproduction systems (autogamy) and of domestication and breeding history [14]. On the contrary, wild tomato species are characterised by a rich genetic variability and can provide plant breeders with a broad pool of potentially useful gene/alleles that could be introduced into modern varieties to improve specific traits. Many tomato cultivars have been improved for quality traits as a consequence of the introgression of wild alleles responsible for increased soluble solid content, fruit colour and adaption to harvesting [15]. Among the available tomato genetic resources, the introgression lines (ILs), which carry homozygous genomic regions of wild tomato species in the cultivated *S*. *lycopersicum* genetic background, can provide a wide array of unexplored genetic variation and represent a valuable tool for breeders [16,17]. Indeed, the introgression lines of *S*. *pennellii* into the genomic background of the cultivated *S*. *lycopersicum* genotype M82 were successfully used to map several QTLs and identify candidate genes associated to fruit quality traits [17–20].

Breeding programmes that include crop wild relatives with lower or higher allergenic potential, may result in cultivars that have a potential of safe or unsafe consumption for affected consumers. In fact, there is a vast array of gene products that are potentially brought from the wild relatives into the cultivated genetic background and that could possibly interact with those in the cultivated crop modifying proteins across allergen pathways [13]. With this in mind, in this study we assessed the allergenicity of two tomato *S*. *pennellii* introgression lines in comparison with the cultivated M82 used as control. We investigated both IL7-3 and IL12-4 since previous studies demonstrated that they could be potentially used for breeding schemes because of their high antioxidant content [21]. In order to assess the allergenic potential of the two ILs we evaluated the positive interactions of IgEs of tomato-allergic subjects and we identified putative tomato allergens using proteomic analyses. In addition, bioinformatic and transcriptomic investigations were carried out to analyse the structure and the expression profiles of the identified allergen encoding genes. Such kind of analyses, focused on the relationship between the introgression of wild alleles and the potential allergenicity of tomato fruits, have never been done before and could help to isolate wild *loci* influencing tomato fruits allergenic properties [22].

## Materials and Methods

### Plant cultivation

Seeds from the *S*. *pennellii* in *S*. *lycopersicum* introgression lines IL12-4 (accession LA4102), IL7-3 (accession LA4066) and from their parental line M82 (accession LA3475) were kindly provided by the Tomato Genetics Resource Centre (TGRC) (http://tgrc.ucdavis.edu/). In the years 2012, 2013 and 2014, tomato plants were grown according to a completely randomized block design with three replicates (10 plants/replicate) in an experimental field located in Acerra (Naples, Italy). Samples of ~20 full mature red fruits *per* plant were collected. Tomato fruits at comparable red-ripe stage were chopped, ground in liquid nitrogen by a blender (FRI150, Fimar) to a fine powder and kept at -80°C until the analyses.

### Tomato allergic subjects

We examined sera from 10 adults (M/F 6/4; age range 18–44 years) with a history of tomato allergy (oral allergy syndrome) hypersensitive to profilin, as shown by positive SPT (skin prick testing) performed with a commercial date palm pollen profilin extract (50 μg/ml; ALK-Abellò, Lainate Italy) and by circulating IgE specific for the grass pollen profilin Phl p 12 measured by ImmunoCAP (Thermo Fisher Scientific, Uppsala, Sweden). Sera from tomato-allergic patients previously used to carry out specific IgE measurements within routine clinical practice and subsequently stored at -20°C in the serum bank of the Allergy Unit of Clinica San Carlo were employed to perform the study. All patients gave their verbal consent to perform the serological investigations. Thus no approval from an Ethics review Board was needed.

Specific IgE levels for profilin ranged between 0.78 and 9.69 KU/L. Values < 0.35 kU/L were considered negative. The sera were pooled to carry out all the immunochemical analyses. The serum pool was aliquoted and stored at -20°C until use.

### Protein extraction preparation

Ripe tomatoes were collected, washed in distilled water and carefully peeled. Peel and pulp were cut in small pieces and lyophilized. Protein extraction was performed from peel and pulp separately. Briefly, 1 gr of tomato tissue was homogenized by means of mortar and pestle in 10 ml of phosphate-buffered saline/polyvinylpolypyrrolidone [PBS/PVPP, 0.15 M NaCl, 0.01 M NaH_2_PO_4_, pH 7.4, 1% (w/w) polyvinylpolypyrrolidone] and complete protease inhibitor cocktail (Sigma-Adrich, Italy). Raw materials were extracted for 3 hours at 4°C under gentle agitation. Then, samples were centrifuged and the supernatants were collected and concentrated. Protein content was determined by the Bradford method (Bradford, 1976) using bovine serum albumin as a standard (Micro-Bio-Rad Protein Assay, Bio-Rad Laboratories, Segrate, Italy).

### Protein slot blotting

Slot blot technique was applied to assess the whole allergenicity of pulp and peel of each tomato line under investigation. Three different experiments per extraction were performed. Equal amounts of protein extracts (2μg/well) were denatured adding 20 mM dithiothreitol (DTT) and boiling the samples for 10 min. Then, they were bound to nitrocellulose membrane and first stained with the Ponceau S staining solution [0.1% (w/v) Ponceau S in 5% (v/v) acetic acid] to check that same amounts of proteins were loaded in each well. Membranes were incubated overnight with pooled sera (diluted 1:10; PBS Tween 0.1%). After washing, membranes were incubated with polyclonal anti-human IgE-secondary antibody conjugated with peroxidase (Sigma-Aldrich, Italy) and the signals were detected using the SuperSignal West Dura detection kit (Pierce). X-ray film (Kodak) image analysis was applied to quantify reactivity signals. Image-Pro Plus (Media Cybernetics) was used to quantify immunochemical signals determining the integral optical density (IOD) of the immunoreactive spots. Means of three independent experiments were calculated and statistically analysed by the GRAPHPAD PRISM program for Windows applying ANOVA and Tukey’s test (p < 0.05).

### Immunoblot and allergen identification

The protein profile of the extracts was evaluated using 4–20% gradient sodium dodecyl sulfate polyacrylamide gel electrophoresis (SDS-PAGE). A total of 20 μg/lane of each extract was loaded. After electrophoresis, gels were stained with a very sensitive colloidal Coomassie G-250 [23] or transferred to nitrocellulose membrane. Successively, nitrocellulose filters were stained with the Ponceau S staining solution [0.1% (w/v) Ponceau S in 5% (v/v) acetic acid] to check that same amounts of proteins were loaded in each well (S1 Fig). Immunoblotting was performed according to Aina et al. (2010) [24]. Nitrocellulose filters were treated as reported for slot blot membranes.

Four bands at 46 kDa, 34 kDa, 23 kDa and 9 kDa, were cut from the SDS-PAGE gel, digested with trypsin and individually identified by LC-MS/MS spectrometry (Liquid Chromatography-Mass spectrometry/mass). Protein identity was assessed by the Global Proteome Machine software (www.thegpm.org) against available *Solanum lycopersicum* protein databases. Proteins were identified by ISB (Ion Source and Biotechnologies S.r.l, Milan, Italy).

### Analysis of glycosylated protein

In order to determine differences among the three tomato lines in the IgE binding to glycosylated allergens, periodate oxidation was performed after proteins were separated by SDS-PAGE gradient gel (4–20%) and transferred onto nitrocellulose membranes. Filters were incubated in 0.3 M sodium acetate, pH 4.5, containing 10 mM sodium metaperiodate for 1h at 20°C in the dark. Filters were then washed and IgE binding capacity was analysed, as reported for slot blot membranes, and compared with those from untreated filters.

### Identification of putative tomato allergens and bioinformatics analyses

*S*. *lycopersicum* gene structure annotations were derived from the GFF3 file downloaded from the SGN ftp server (ftp://ftp.solgenomics.net/genomes/Solanum_lycopersicum/annotation/ITAG2.4_release/ITAG2.4_gene_models.gff3). The Fancy GENE tool was used for drawing pictures of gene structures (http://bio.ieo.eu/fancygene/; [25]). *S*. *pennellii* gene structure annotations were derived from the GFF3 file downloaded from the SGN ftp server (ftp://ftp.solgenomics.net/genomes/Solanum_pennellii/spenn_v2.0_gene_models_annot.gff). The ends of the introgression were mapped exploiting data reported in the following file (ftp://ftp.solgenomics.net/genomes/Solanum_pennellii/sgnMarkersSpenn.gff3). For the prediction of glycosylation sites within amino acid sequences we used the GPP Prediction Server at http://comp.chem.nottingham.ac.uk/cgi-bin/glyco/bin/getparams.cgi. [26]. In order to represent the glycosylation sites on the protein sequence we used the tool at http://prosite.expasy.org/mydomains.

### Real-time PCR

Total RNA was isolated from peel and pulp from tomato fruit of M82, IL7-3 and IL12-4 using the TRIzol^®^ reagent (Invitrogen, Carlsbad, CA, USA) and treated with the RNase-free DNase Set (Invitrogen, Carlsbad, CA, USA Madison, WI, USA) according to the method reported by the manufacturer (Invitrogen). Total RNA (1 μg) was reverse transcribed using the Transcriptor High Fidelity cDNA Synthesis Kit (Roche) and cDNA was stored at -20°C until RT-PCR analysis. One μL of the resulting cDNA diluited 1:10 was mixed with 12.5 μL SYBR Green PCR master mix (Applied Biosystems, Warrington, UK) and 5 pmol each of the forward and reverse primers (listed in S1 Table) in a final volume of 25 μL. The reaction was carried out using the 7900HT Fast-Real Time PCR System (Applied Biosystems, Warrington, UK). The amplification program included the following steps: 2 min at 50°C, 10 min at 95°C, 0.15 min at 95°C and 60°C for 1 min for 40 cycles, followed by the thermal denaturing step (0.15 min at 95°C, 0.15 min at 60°C, 0.15 min at 95°C) to generate the dissociation curves in order to verify the amplification specificity. Relative RNA accumulation was calculated using the elongation factor 1- α (Solyc06g005060) as reference gene. All reactions were run in triplicate for each of the three biological replicates. Comparison of RNA expression was based on a comparative CT method (ΔΔCT) and the relative expression was quantified and expressed according to RQ calculated as 2^−ΔΔCT^, where ΔΔCT = (CT _RNA target_ − CT _reference RNA_) − (CT _calibrator_ − CT _reference RNA_) [27]. M82 pulp was selected as calibrator for the analysed genes. Quantitative results were expressed as mean values ± SE. Differences among samples were determined by using SPSS (Statistical Package for Social Sciences) Package 6, version 15.0. Significance was determined by comparing the samples through a *t*-Student’s test at a significance level of 0.05.

## Results and Discussion

In this study the allergenicity of two tomato *S*. *pennellii* introgression lines (namely, IL7-3 and IL12-4) in comparison to the parental line (the cultivated genotype M82) was assessed in order to relate the introgression of wild genes with the potential allergenicity of tomato fruits. This work was carried out by using a multidisciplinary approach that included proteomic, bioinformatic and transcriptomic investigations in order to assess the allergenicity of the lines under study and to analyse the structure and the expression of the genes encoding for the identified allergens.

### Assessment of total allergenicity

**Fig 1:**
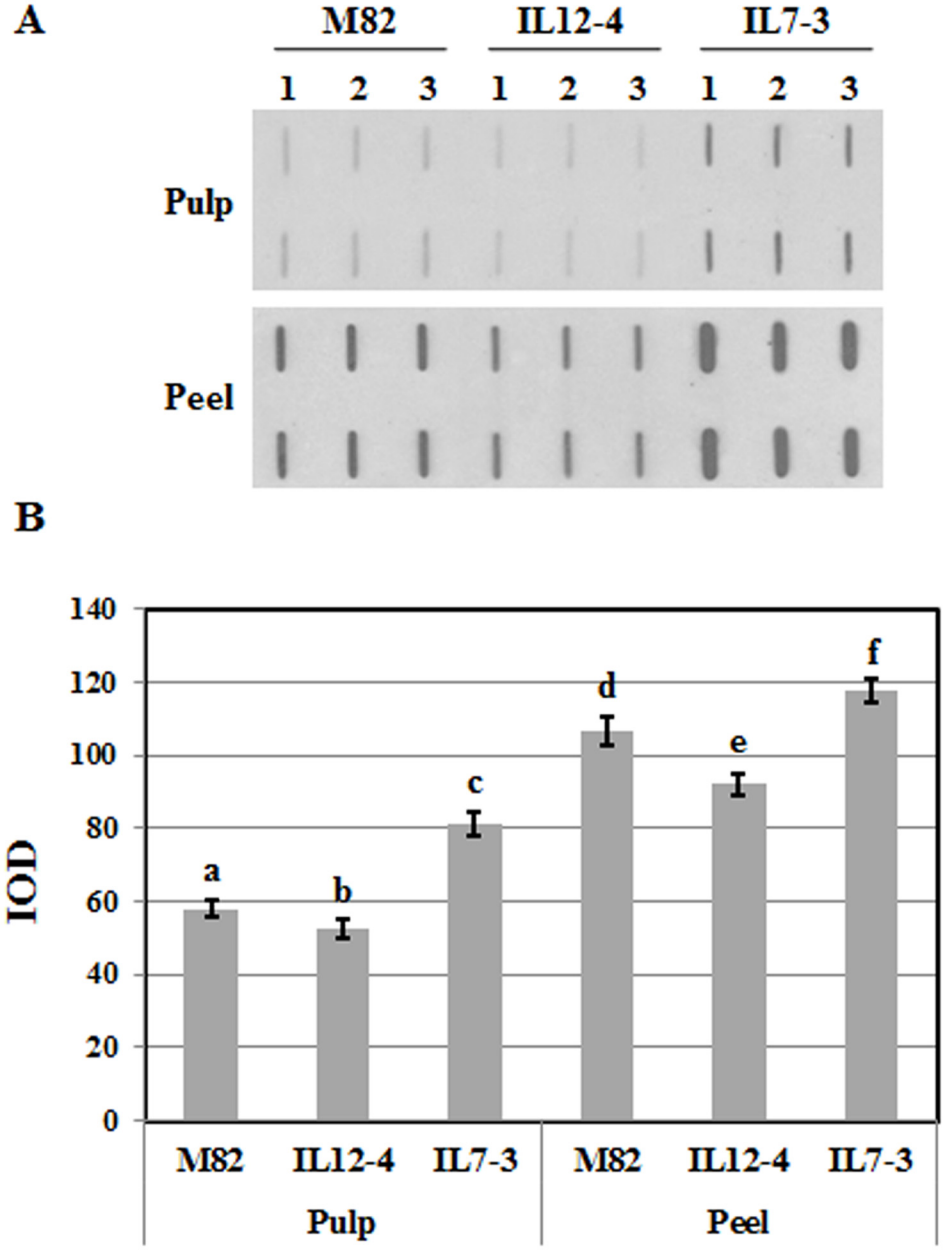
Tomato pulp and peel total allergenicity in IL7-3, IL12-4 and in the cultivated line M82 assessed by slot blotting. (A) Example of a X-ray film obtained by slot blot analyses using pooled sera of allergic patients; three different replicates for each line were analysed. (B) Assessment of total allergenicity, through image analysis, measuring integrated optical density (IOD) of immunoreactive bands. Different letters indicate significant differences among the samples (ANOVA and Tukey’s test, p < 0.05). See text for details.

The total allergenicity of the two introgression lines and of the cultivated line M82 was assessed by slot blotting. Panel A in Fig 1 shows an example of filters after slot blot and immunodetection. These analyses highlighted a difference between the allergenic potential of the pulp and the peel for all the lines under investigation. In particular, pooled sera of tomato allergic patients were mostly immunoreactive to proteins extracted from the peel compared to the pulp. This result is in accordance with already available data on the allergenicity of Rosaceae fruits, such as apple, peach and pear [27, 28]. The overall total allergenicity was very different in IL7-3 and IL12-4 compared to the parental line M82. In fact, higher IgE reactivity was detected for both pulp and peel tissues from IL7-3 fruits. This line harboured a statistically higher allergenic potential compared to the cultivated genotype M82 and also to IL12-4 (Fig 1B). By contrast, the IgE binding activity of IL12-4 was less marked, showing a tendency to a reduced allergenicity compared to the other two genotypes, particularly when samples extracted from the peel were taken into account (Fig 1B).

### Identification of tomato allergens

**Fig 2:**
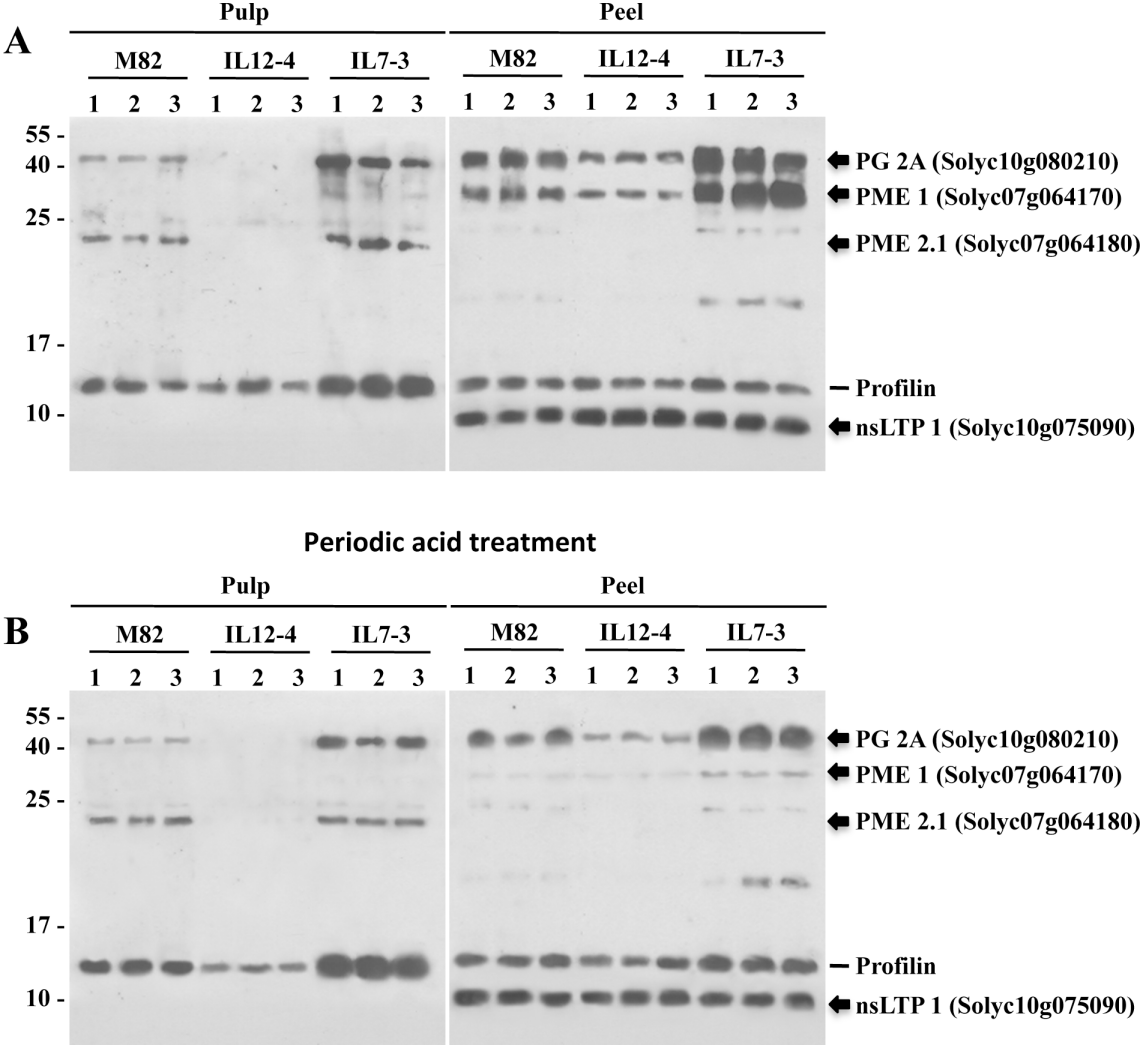
Putative allergens identified on immunoblots with extracts from fruits from M82, IL12-4 and IL7-3. (A) Protein extracts from three replicates of each line were analysed by immunoblotting using sera of tomato allergic subjects. Protein bands that showed a stronger reaction (black arrows) were eluted from the gels and identified using mass spectrometry. PG2A (Polygalacturonase 2A) Solyc10g080210; PME1 (Pectin methylesterase 1) Solyc07g064170; PME2.1 (Pectin methylesterase 2.1) Solyc07g064180; nsLTP 1 (non-specific lipid transfer protein type 1) Solyc10g075090. (B) Membranes were treated with periodic acid before immunoblotting with allergic patient sera. A 14 kDa protein (Profilin) detected in immunoblots is also shown.

Immunoblot analyses with pooled sera of 10 tomato allergic subjects were conducted to identify putative tomato allergens present in the peel and in the pulp of tomato fruits from M82, IL7-3 and IL12-4. Several bands in a molecular mass ranging from 6 to 50 kDa reacted with the sera IgE (Fig 2A).

The allergenic profile, as determined by pooled sera, showed some differences among the three lines. A protein of 46 kDa was detected in the samples extracted from the peel for all the lines and only in the pulp of IL7-3 and M82 fruits. A protein of 34 kDa was detected in all the samples extracted from the tomato peel. By contrast, a protein of 23 kDa was identified only in extracts from the tomato pulp of IL7-3 and M82 fruits. Other two proteins, of 18 and 9 kDa respectively, were observed only in the peel. Interestingly, the protein of 18 kDa was never detected in extracts from the IL12-4 line. Finally, a protein of 14 kDa was recognized in all the analysed samples, probably corresponding to a profilin, as we used a pool of sera from profilin hypersensitive patients.

Among the proteins that reacted with the serum pool, four putative allergens chosen on the basis of the intensity of the reaction and on the presence in the different lines, were further analysed by mass spectrometry. These analyses led to the identification of the 46 kDa protein as the polygalacturonase 2A (PG2A; Solyc10g080210; EC 3.2.1.15). The 34 kDa and 23 kDa proteins corresponded to the pectin methylesterases 1 and 2.1, respectively (PME1 and PME2.1; Solyc07g064170 and Solyc07g064180; EC 3.1.1.11) and the 9 kDa protein corresponded to a type 1 non-specific lipid transfer protein (nsLTP; Solyc10g075090; EC2.3.1.176) (Table 1). All the identified proteins have already been described as putative tomato allergens [29,30]. In particular, the nsLTP corresponds to the already described Sola l 3. The identification of the allergen-encoding genes was reliable due to high sequence coverage and scores with the corresponding database entries obtained as a consequence of BLAST searches. The theoretical molecular weights of the identified proteins not always were in agreement with the values experimentally achieved; however, these differences, which were observed also in other studies [12], could be due to partly processed proteins.

**Table 1:** Features of the allergen-encoding genes identified by mass spectrometry.

Name	Gene ID	Gene coordinates	Protein length (num.aa)	Molecular weight (kDa)	Number of glycosylated residues
Pectin methylesterase 1 (PME1)	Solyc07g064170	66430156..66432717	547	60.08	35
Pectin methylesterase 2.1 (PME2.1)	Solyc07g064180	66440014..66443271	551	60.51	36
Non-specific lipid transfer protein (nsLTP)	Solyc10g075090	58800507..58801024	122	12.21	14
Polygalacturonase 2A (PG2A)	Solyc10g080210	61559168..61565803	458	50.06	50

Bioinformatic analyses were adopted for predicting the locations and the exon-intron structures of genes in the tomato genome. The structures of the two PME-encoding genes, of the PG2A and of the nsLTP are reported in S2 Fig. None of the genes fall within the reported introgressed regions 7–3 and 12–4, even if the two PME-encoding genes are located immediately down the bottom edge of the introgressed region.

### Glycosylation analyses

**Fig 3:**
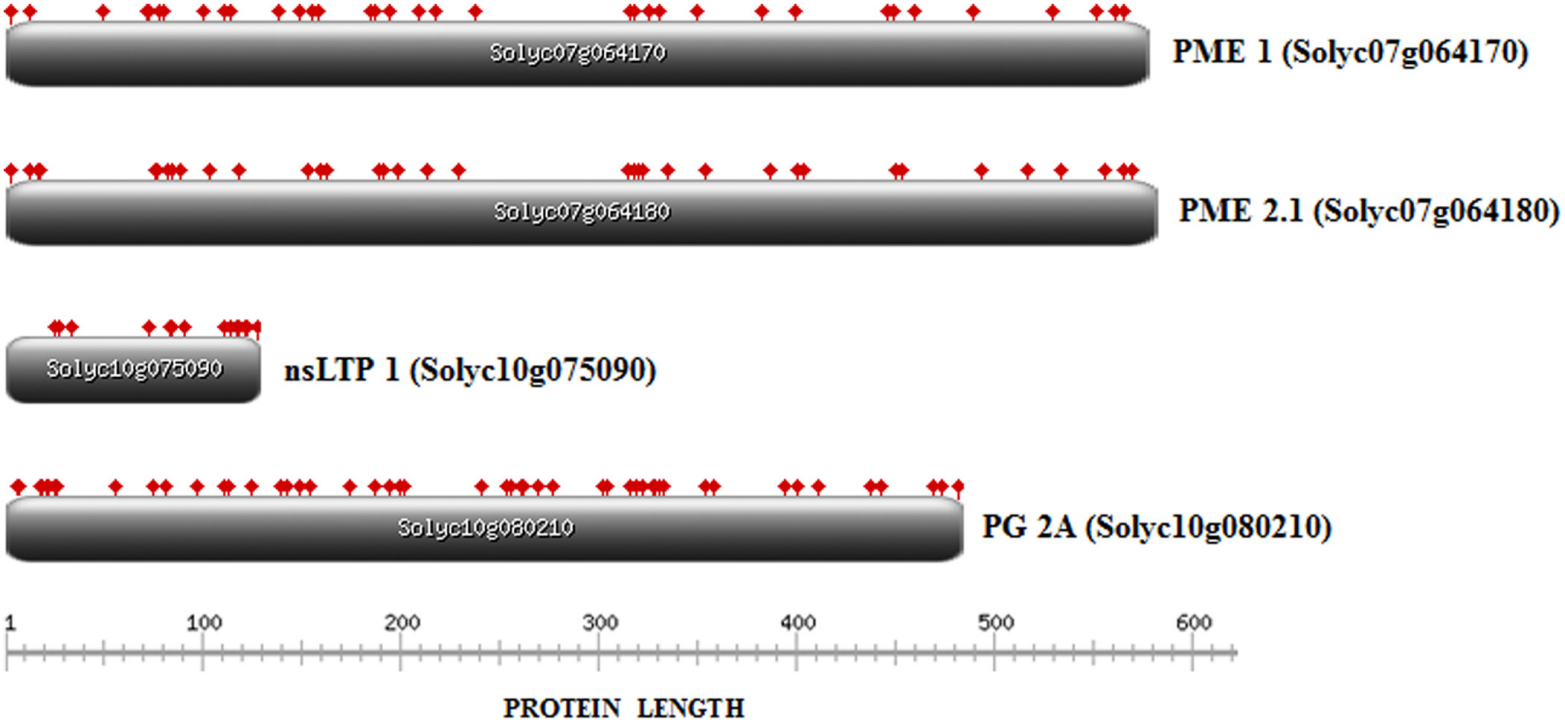
Predicted glycosylation sites on the allergen encoding genes Solyc07g064170 (PME1), Solyc07g064180 (PME2.1), Solyc10g075090 (nsLTP) and Solyc10g080210 (PG2A) represented by flag-tags along the protein sequences. For the prediction of glycosylation sites within amino acid sequences the GPP Prediction Server at http://comp.chem.nottingham.ac.uk/cgi-bin/glyco/bin/getparams.cgi was used. The glycosylation sites on the protein sequence were represented using the tool at http://prosite.expasy.org/mydomains.

Some tomato allergens are glycoproteins comprising N-glycan structures (mainly β(1,2)-xylose and α(1,3)-fructose) also called cross-reactive carbohydrate determinants (CCD). It is known that some allergic subjects possess specific IgE antibodies against these CCDs [3]. In particular, the PME and PG proteins, which have been here identified as putative tomato allergens, are known to own N-glycosylation sites [12,31]. N-linked as well as O-linked glycosylation sites were predicted and annotated for all the proteins under investigation. As it is evident from Fig 3, the distribution of potential glycosylation sites is variable across the allergens even if the two PMEs (Solyc07g064170 and Solyc07g064180) include almost the same number of glycosylation sites, as expected. In addition, we analysed the wild introgressed region 7–3 (that spans 32 cM along chromosome 7) and the wild introgressed region 12–4 (that spans 52 cM along chromosome 12) and demonstrated that both regions include several genes encoding glycosyltransferase enzymes that catalyse the formation of the glycosidic linkage on proteins (S2 Table). However, a higher number of these genes were found on the introgressed region 7–3; in addition, immediately downstream the bottom edge of the introgressed region 7–3 and following the positions of the two PMEs, it is located a gene coding for a β(1,2)-xylosyltranferase (Solyc07g065140). Noteworthy, previous studies demonstrated that the silencing of the Solyc07g065140 gene in transgenic tomato plants resulted in fruits with a lower allergenic potential compared to untransformed samples [32].

Based on these evidences we decided to investigate if the differences in allergenicity among the ILs and M82 were due also to differences in IgE recognition of glycosylated proteins. Our results demonstrated that Ig-E binding capacity of the tomato allergens decreased after periodate treatment (Fig 2B). In particular, this treatment led to an important reduction of IgE binding to the PME1 allergen (34 kDa) in all samples, indicating that carbohydrates represent the IgE binding determinants or are involved in the conformation of allergenic epitopes in this allergen. This phenomenon was more dramatic in the peel of the tomato fruits for all the tested lines. Immunoblot data (Fig 2B) indicate that the different allergenicity demonstrated by the three tomato lines may be linked also to differences in the N-glycan structure of the allergenic proteins in the two introgression lines compared to M82.

### Expression of allergen-encoding genes

Expression profiles of the allergen-encoding genes in the pulp and peel tissues of tomato fruits harvested from the three lines were assessed by using qRT-PCR. This was done in order to understand if the allergenic potential of the tomato genotypes was related to variation in the expression level of allergen-encoding genes.

These analyses demonstrated that the gene coding for the nsLTP protein (Solyc10g075090) was up-regulated in the peel compared to the pulp in all the lines. In addition, this gene showed a lower RNA accumulation in the peel of the ILs and in the pulp of the IL12-4 compared to M82. This result is in good agreement with results obtained by immunoblot analyses in which the nsLTP protein was recognized only in peel extracts in all the lines. The gene Solyc10g80210 coding for the protein PG2A resulted up-regulated both in IL7-3 and IL12-4 peels compared to M82, and showed higher expression in the peel compared to the pulp in all the lines. The gene coding for the two PMEs (Solyc07g064170 and Solyc07g064180) were up-regulated in the pulp of all the introgression lines compared to M82.

**Fig 4:**
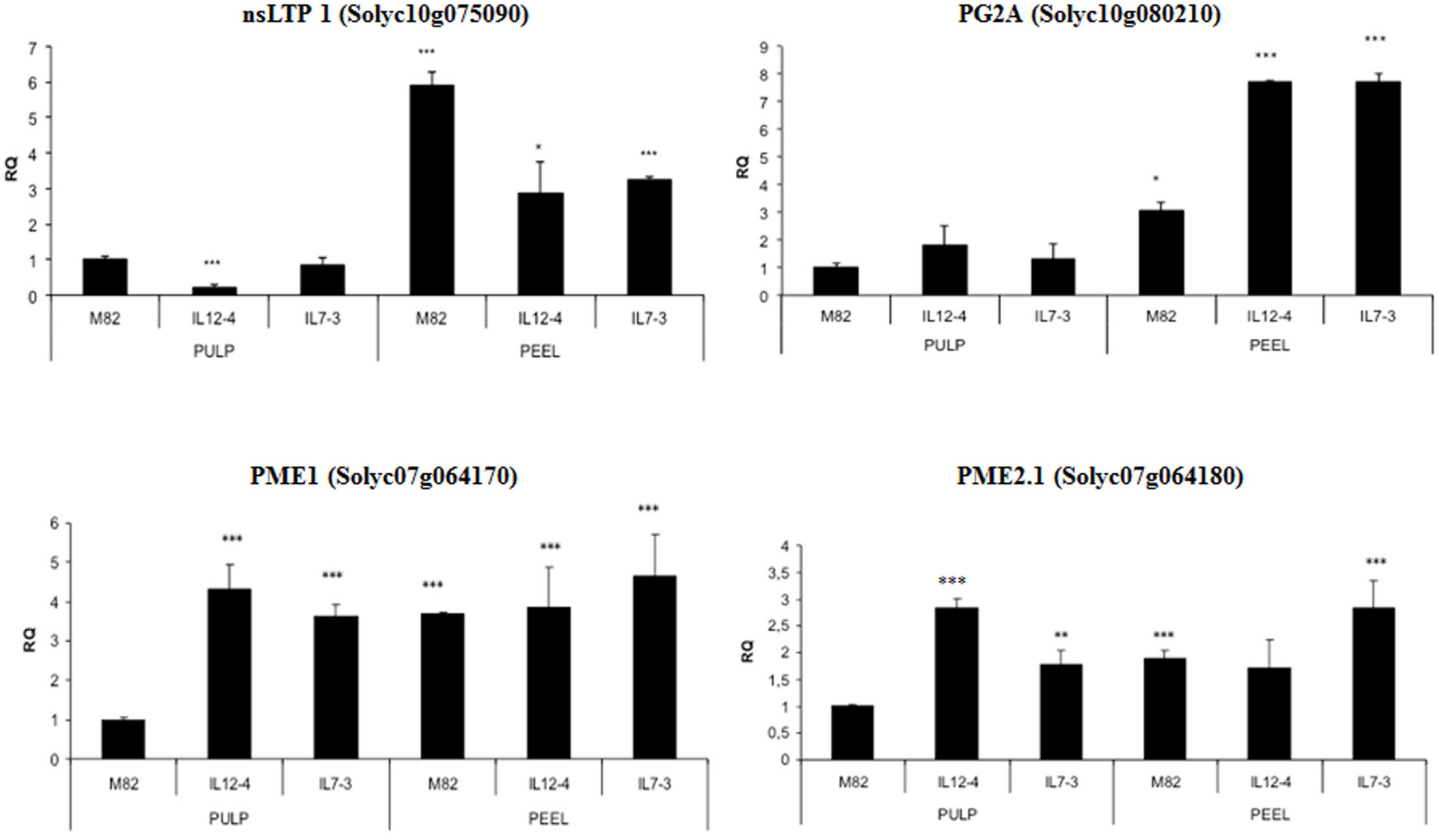
Relative RNA accumulation of allergen-encoding genes in the pulp and the peel of tomato fruits from the lines IL12-4, IL7-3 and M82. qRT-PCR analyses were carried out with primers pairs for the genes Solyc10g075090 (nsLTP 1), Solyc10g080210 (PG2A), Solyc07g064170 (PME1), Solyc07g064180 (PME2.1) using an elongation factors (Solyc06g005060) as endogenous control. The transcript amount in the pulp of M82 fruit was arbitrarily set to 1 and it was used as calibrator for relative expression levels in each comparison. Asterisks indicate values that are significantly different from M82 pulp (*p < 0.05, ** p < 0.01, *** p < 0.001).

Altogether, RT-PCR analyses (Fig 4) demonstrated that the differences observed in immunoblot analyses for the PG2A and the two PMEs were independent of RNA accumulation of the allergen-encoding genes. As a consequence, such differences were likely due to dissimilarities in the expression pattern not measurable by qRT-PCR or most probably can be ascribed to line-dependent translational control or post-translational modifications of the corresponding proteins, which are crucial for determining their allergenic potential.

## Conclusion

Previous studies have shown that the choice of tomato cultivar significantly influences the allergic reaction of tomato allergic subjects [2]. This work aims to address the question of whether the introgression of genomic regions from the wild species *S*. *pennellii* in the genomic background of cultivated tomato line (M82) may influence the allergenic potential of tomato fruits. It points out that when this species is used in breeding schemes to transfer favourable alleles in cultivated tomato varieties, it is also crucial to assess the risk linked to the potential allergenicity of the new-developed plant material. Indeed, we identified the PG2A (Solyc10g080210), the PME1 (Solyc07g064170) and PME2.1 (Solyc07g064180), the nsLTP (Solyc10g075090) and the profilin as the main allergens present in tomato fruits. In addition, we confirmed the different allergenic potential of the pulp and the peel, with this latter showing a higher allergenic potential.

We showed that there are genomic regions from wild tomato species, such as *Solanum pennellii*, that may deeply influence the allergenic potential of tomato fruits. Indeed, fruits harvested from the introgression line IL7-3 harbour a higher total allergenic potential compared to those from the cultivated genotype M82 and from IL12-4. On the contrary, lower IgE-mediated allergic reactions were observed in extracts of IL12-4 compared to IL7-3 and M82. The variability in the allergenic potential among the three tomato lines was mostly due to differences in IgE recognition patterns of a PG2A (46 kDa), one of the major tomato allergen, and of a PME1 (34 kDa) that were more immunoreactive in IL7-3 compared to IL12-4 and M82.

We demonstrated that the allergenicity of tomato fruits is not always linked to different expression levels of the allergen-encoding genes, rather it relays on the line-dependent translational control or post-translational modifications. In particular, analyses carried out in this study suggest that the allergenicity of tomato fruits may be associated to different glycan structures of the identified proteins. Now, further studies aiming at dissecting the two ILs into sub-lines that carry introgressed regions of reduced size compared to the existing ones are underway. It is very likely that these efforts will contribute to isolate, in a more accurate way, the favorable wild genes responsible for the low allergenic potential of the region 4 on chromosome 12. At the same time, the identification of the unfavorable wild genes that determine the high allergenic potential of the region 3 on chromosome 7 can be achieved.

Altogether, the identification of genetic resources with different allergenic potential represents a valuable tool both to understand the molecular basis of tomato allergic disease and for the development of novel hypoallergenic varieties. Allergic subjects could benefit from the development of such hypoallergenic tomato fruits since they could avoid eliminating completely this important crop from their diet, increasing their quality of life. However, it is worth saying that the potential hypoallergenicity of tomatoes cannot be generalised for all the allergic subjects, since it has to be assessed according to the specific allergenic protein to which every single allergic subject is sensitised [8].

## Supporting Information

S1 Fig Protein profile of the extracts from the pulp and the peel of tomato fruits from the lines M82, IL12-4 and IL7-3 evaluated using 4–20% gradient sodium dodecyl sulfate polyacrylamide gel electrophoresis (SDS-PAGE). (A) Staining of the nitrocellulose membrane used for subsequent immunoblot analyses with Panceu S (B) Staining of loaded gels with comassie brillant blue.
(TIF)

S2 Fig Exon-intron structures and genomic coordinates of the allergen encoding genes. Black boxes and strand lines represent exons and introns, respectively. The 5′ to 3′ orientation is indicated by the arrow in the last exon.
(TIF)

S1 Table List of primers used for Real-time PCR experiments. (PDF)

S2 Table List of genes encoding glycosyltransferase enzymes that catalyze the formation of the glycosidic linkage on proteins in the introgression regions 7–3 and 12–4. (PDF)
