# Supplementary figures and images for: Impact of Wild Loci on the Allergenic Potential of Cultivated Tomato Fruits
Source: PLoS One. 2016 May 16;11(5):e0155803. doi: 10.1371/journal.pone.0155803 (PMC4868316; doi:10.1371/journal.pone.0155803)

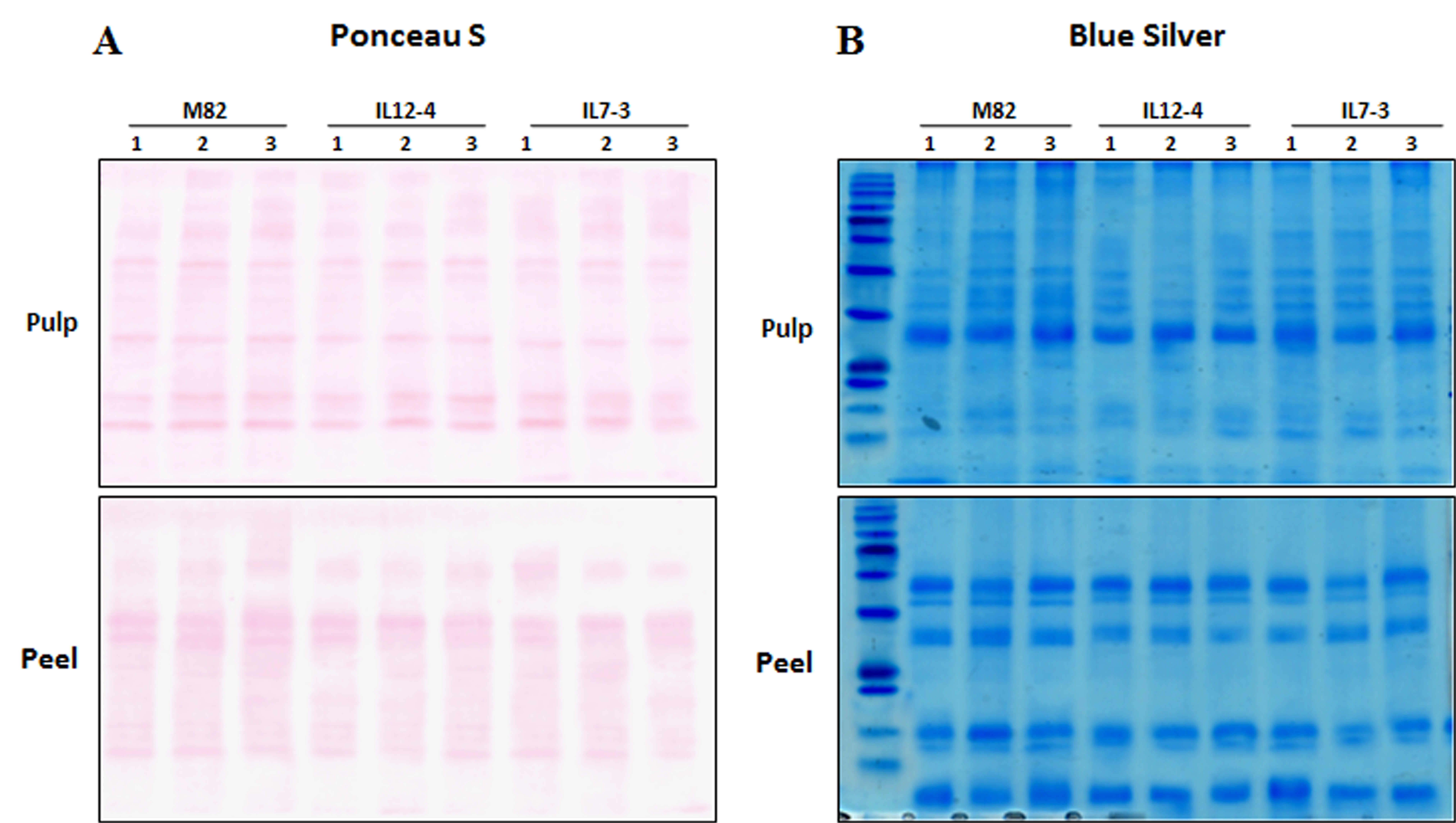

Supplement: S1 Fig — (A) Staining of the nitrocellulose membrane used for subsequent immunoblot analyses with Panceu S (B) Staining of loaded gels with comassie brillant blue. (TIF) [file pone.0155803.s001.tif]
