# Supplementary material for: Impact of Wild Loci on the Allergenic Potential of Cultivated Tomato Fruits
Source: PLoS One. 2016 May 16;11(5):e0155803. doi: 10.1371/journal.pone.0155803 (PMC4868316; doi:10.1371/journal.pone.0155803)
Supplement: S1 Table — (PDF) [file pone.0155803.s003.pdf]

S1 Table. List of primers used for Real-time PCR experiments.

Annotation	gene ID	Sequence
Pectinesterase 1	Solyc07g064170	FW: 5’-CTTGGGTGAGTTCGATGGATAG-3’
	Solyc07g064170	RV: 5’-CGTCTTACTCTTATCTGGTGCTG-3’
Pectinesterase	Solyc07g064180	FW: 5’-GAGAGTTCGGGTAAGGACATTG-3’
	Solyc07g064180	RV: 5’-CGTCTTACTCTTATCTGGTGCTG-3’
Non-specific lipid-transfer protein 2	Solyc10g075090	FW: 5’-TGTTAAGGGTCTATTGGGCG-3’
	Solyc10g075090	RV: 5’-GCGGCTTTTCCTGTATCAATG-3’
Polygalacturonase 2A	Solyc10g080210	FW: 5’-CATTTGGTAGTGTTTCTTATCGTGT-3’
	Solyc10g080210	RV: 5’-TTTAGGAACCACAAATTGAACAGG-3’
Elongation factor	Solyc06g005060	FW: 5’-CAACCCTGACAAAATCCCCTTT-3’
	Solyc06g005060	RV: 5’-TTGGTCCCTTGTACCAGTCGAG-3’
