# Supplementary material for: Impact of Wild Loci on the Allergenic Potential of Cultivated Tomato Fruits
Source: PLoS One. 2016 May 16;11(5):e0155803. doi: 10.1371/journal.pone.0155803 (PMC4868316; doi:10.1371/journal.pone.0155803)
Supplement: S2 Table — (PDF) [file pone.0155803.s004.pdf]

S2 Table. List of genes encoding glycosyltransferase enzymes that catalyze the formation of the glycosidic linkage on proteins in the introgression regions 7-3 and 12-4.

***S. lycopesicum* gene ID**	***S. pennellii* gene ID**	**Chromosome**	**Gene**
Solyc07g049310.2.1	Sopen07g024640.2	7	D-xylosetransporter
Solyc07g049610.1.1	Sopen07g025010.1	7	Galactosyltransferase-likeprotein (Fragment)
Solyc07g052320.2.1	Sopen07g025940.1	7	Beta-1 3-galactosyltransferase 6
Solyc07g052630.1.1	Sopen07g026240.1	7	Glycosyltransferase-likeprotein
Solyc07g052640.2.1	Sopen07g026240.1	7	Glycosyltransferase-likeprotein
Solyc07g052650.1.1	Sopen07g026250.1	7	Glycosyltransferase-likeprotein
Solyc07g053920.2.1	Sopen07g027900.1	7	Mannan endo-1 4-beta-mannosidase
Solyc07g053980.2.1	Sopen07g027970.1	7	Glucansynthaselike1
Solyc07g054440.2.1	Sopen07g028370.1	7	Beta-1,3-galactosyl-O-glycosyl-glycoprotein beta-1,6-N-acetylglucosaminyltransferase 7
Solyc07g055930.2.1	Sopen07g029290.1	7	Glycosyltransferase
Solyc07g055990.2.1	Sopen07g029340.1	7	Xyloglucanendotransglucosylase/hydrolase7
Solyc07g056000.2.1	Sopen07g029350.1	7	Xyloglucanendotransglucosylase/hydrolase7
Solyc07g056260.2.1	Sopen07g029620.1	7	Glucansynthaselike
Solyc07g062540.2.1	Sopen07g030770.1	7	Alpha-1 2-mannosyltransferase
Solyc07g062590.2.1	Sopen07g030820.1	7	Beta-1 3-galactosyltransferase 6
Solyc07g063110.2.1	Sopen07g031300.1	7	Beta-1,3-galactosyl-O-glycosyl-glycoprotein beta-1,6-N-acetylglucosaminyltransferase
Solyc12g096440.1.1	Sopen12g032280.1	12	Beta-1 3-galactosyltransferase 6
Solyc12g099680.1.1	Sopen12g034370.1	12	Glycosyltransferase-likeprotein
Solyc12g099730.1.1	Sopen12g034370.1	12	Glycosyltransferase-likeprotein
